# Comparison of Proposed Modified and Original Sequential Organ Failure Assessment Scores in Predicting ICU Mortality: A Prospective, Observational, Follow-Up Study

**DOI:** 10.1155/2016/7379325

**Published:** 2016-12-25

**Authors:** Afshin Gholipour Baradari, Hassan Sharifi, Abolfazl Firouzian, Maryam Daneshiyan, Mohsen Aarabi, Yaser Talebiyan Kiakolaye, Seyed Mahmood Nouraei, Alieh Zamani Kiasari, Mohammad Reza Habibi, Amir Emami Zeydi, Faegheh Sadeghi

**Affiliations:** ^1^Department of Anesthesiology, Faculty of Medicine, Mazandaran University of Medical Sciences, Sari, Iran; ^2^Department of Medical-Surgical Nursing, School of Nursing and Midwifery, Iranshahr University of Medical Sciences, Iranshahr, Iran; ^3^Department of Epidemiology, Faculty of Medicine, Mazandaran University of Medical Sciences, Sari, Iran; ^4^Critical Care Nursing, Cancer Institute, Tehran University of Medical Sciences, Tehran, Iran; ^5^Department of Cardiac Surgery, Faculty of Medicine, Mazandaran University of Medical Sciences, Sari, Iran; ^6^Department of Medical-Surgical Nursing, Faculty of Nursing and Midwifery, Mazandaran University of Medical Sciences, Sari, Iran; ^7^Faculty of Medicine, Mazandaran University of Medical Sciences, Sari, Iran

## Abstract

*Background*. The sequential organ failure assessment (SOFA) score has been recommended to triage critically ill patients in the intensive care unit (ICU). This study aimed to compare the performance of our proposed MSOFA and original SOFA scores in predicting ICU mortality.* Methods*. This prospective observational study was conducted on 250 patients admitted to the ICU. Both tools scores were calculated at the beginning, 24 hours of ICU admission, and 48 hours of ICU admission. Diagnostic odds ratio and receiver operating characteristic (ROC) curve were used to compare the two scores.* Results*. MSOFA and SOFA predicted mortality similarly with an area under the ROC curve of 0.837, 0.992, and 0.977 for MSOFA 1, MSOFA 2, and MSOFA 3, respectively, and 0.857, 0.988, and 0.988 for SOFA 1, SOFA 2, and SOFA 3, respectively. The sensitivity and specificity of MSOFA 1 in cut-off point 8 were 82.9% and 68.4%, respectively, MSOFA 2 in cut-off point 9.5 were 94.7% and 97.1%, respectively, and MSOFA 3 in cut-off point of 9.3 were 97.4% and 93.1%, respectively. There was a significant positive correlation between the MSOFA 1 and the SOFA 1 (*r*: 0.942), 24 hours (*r*: 0.972), and 48 hours (*r*: 0.960).* Conclusion*. The proposed MSOFA and the SOFA scores had high diagnostic accuracy, sensitivity, and specificity for predicting mortality.

## 1. Introduction

Organ dysfunction and/or failure is one of the main causes of mortality and morbidity in patients hospitalized in the intensive care unit (ICU) [1, 2]. Prediction of mortality and morbidity in the ICUs can enhance clinical decision-making [3], facilitate classifying the critically ill patients according to their health status [4], and improve the quality of provided services for critically ill patients [5, 6].

Several methods for predicting critically patient outcomes are available, including sequential organ failure assessment (SOFA) [7], Acute Physiology and Chronic Health Evaluation (APACHE) [8], Simplified Acute Physiology Score (SAPS), and the Multiple Organ Dysfunction Score (MODS) [9]. The SOFA is one of the useful tools for evaluating organ dysfunction and failure as well as predicting mortality rate in critically ill patients [9].

Previously, the accuracy of SOFA has been determined by several studies and predictive power of this tool has been proven in the critically ill patients with various diseases hospitalized in the ICU [1, 7, 10]. Some studies have reported that the discriminatory power and calibration of the SOFA is higher than the other tools such as Acute Physiology and Chronic Health Evaluation (APACHE) and Simplified Acute Physiology Score (SAPS) [11, 12]. In addition, the ability to review data on a daily basis or every 24 hours in patients is another advantages, whereas most of the other tools are applicable only in the first 24 hours [6, 10]. However, some variables of the SOFA score are expensive or unavailable in some hospitals. The cost of performing SOFA is high and requires specific laboratory equipment that is scarce in some hospitals [13, 14]. To reduce their costs, many hospitals use the traditional methods for evaluating the patient's outcomes such as ICU mortality and morbidity [13]. For this reason, some researchers designed and developed the modified version of SOFA (MSOFA) score as a cost-effective alternative with easy application for predicting mortality in ICU [14, 15].

In the present study, we have proposed another slightly modified version of original SOFA (proposed MSOFA) according to its recognized advantages and disadvantages. Therefore, our study aimed to compare the performance of our proposed MSOFA and original SOFA scoring systems in predicting ICU mortality.

## 2. Methods

This prospective observational study was conducted in the ICU of 600-bed Imam Khomeini hospital, Mazandaran University of Medical Sciences, Sari, Iran, during December 2014 to March 2015. Approval for the project was obtained from the University Ethics Committee.

### 2.1. Eligibility Criteria

All patients older than 18 years with more than 24 hours hospitalization were included. Because of false increased ICU mortality rate, all patients with limits on life-sustaining interventions were excluded, for example, patients who died in less than 24 hours of ICU admission or received CPR before ICU admission. Patients were followed up until ICU discharge in order to document their survival status. ICU nursing staff and physicians were trained theoretically and practically how to calculate and document the proposed modified and original SOFA scores.

### 2.2. Data Collection

Data was collected by research assistances (ICU nursing staff and physicians) in three stages at eight o'clock every morning. The worst physiological score of each organ was calculated using original SOFA and MSOFA at three time series (from number 1 to 3): at the beginning, 24 hours after ICU admission, and then 48 hours after ICU admission. The key variables in this study were main causes of hospitalization in the ICU, length of stay in ICU and hospital outcome, proposed MSOFA and original SOFA scores, and chart of recorded scores for the organs.

### 2.3. Tools

The original SOFA score assesses the severity of disease and evaluates the function of 6 body vital organs on a daily basis [7]. The criteria are as follows: respiratory system with the PO_2_/FiO_2_; cardiovascular system with mean arterial pressure (MAP) and the amount of drug needed to increase blood pressure; coagulation system by measuring platelet counts; liver system by measuring total bilirubin level; nervous system by measuring the Glasgow Coma scale; and renal system with urinary output and creatinine levels. Each organ scores from zero to four, and the total score for all organs calculated from zero to 24. Scores 1-2 determine organ dysfunction while scores 3-4 represent organ failure. The scores were evaluated every 24 hours. If each variable were repeated several times during 24 hour, the worst score would be recorded. After scoring each organ, the total SOFA score was calculated for all patients.

Our proposed MSOFA is derived from the original SOFA score and it has some advantages. MSOFA does not require specific laboratory equipment, is calculated easily on the patient bedside, and is available for daily reassessment. It has six different criteria for each organ, including the respiratory, coagulation, cardiovascular, liver, nervous, and renal. In the following, we describe the modifications. (1) The respiratory system was evaluated by SPO_2_/FiO_2_ criteria. SPO_2_ was obtained using pulse oximetry, and FiO_2_ calculated by the percentage of oxygen adjusted in the ventilator or the percentage of oxygen delivered to the patient by airway. SPO_2_/FiO_2_ is shown as a decimal number in the denominator; for example, FiO_2_ = 55% is written as 0.55. (2) The coagulation system was evaluated with clinical assessment with a score of two for petechia, purpura, and ecchymosis, and a score of four for spontaneous bleeding. (3) The cardiovascular system was assessed by mean atrial pressure (MAP) and the need for vasoactive drugs. If MAP is less than 70 mmHg, vasoactive drugs such as dopamine, dobutamine, epinephrine, and norepinephrine would be prescribed in accordance with hospital policy charting system. Scoring is done based on the required dose of the drug for preserving MAP more than 70 mmHg. (4) The function of liver system was scored by clinical assessment of jaundice with a score of two for jaundice in the sclera and a score of four for jaundice in the skin. (5) The central nervous system was assessed by GCS level. (6) The renal system was assessed by urinary output level based on cc/kg/h.

### 2.4. Study Outcome

The main outcome of the present study was evaluating the sensitivity, specificity, positive, and negative predictive values of our proposed modified SOFA for predicting ICU mortality and disability of the patient until discharge from the ICU and compare these values with the original SOFA scores.

### 2.5. Data Analysis

All statistical analysis was performed using the Statistical Program for Social Sciences (SPSS ver. 18) software. We used descriptive statistics to determine the characteristics of the sample and to describe the study variables. Data were presented as mean + SD, when indicated. Pearson correlation coefficients were calculated to quantify associations between SOFA and MSOFA. Diagnostic odds ratio and ROC curve were used to compare both modified and original scores for sensitivity, specificity, positive, and negative predictive values for the prediction of mortality and disability. The ability of the models for predicting ICU mortality was determined by examining their discrimination, which is the ability of a model to distinguish between a patient who will live and one who will die. Discrimination and/or accuracy was measured by examining the area under the receiver operating characteristics curve (AUC). An area of 1 represents a perfect test; an area of .5 represents a worthless test. The curves were constructed by computing the sensitivity and specificity of increasing numbers of clinical findings (from 0 to 1) in predicting mortality. The model has good discrimination when the AUC is more than 0.8. The difference in the AUC was analyzed by the *z* statistic. The significance level was set at 0.05, and 95% confidence interval was calculated for all of cases.

## 3. Results

### 3.1. Patients Characteristics

A total of 250 patients were included (mean age 45.97 ± 19.46 years, ranged 18–82). There was no statistically difference between men and women age (*P* = 0.106).  Table 1 depicts some characteristics of patients.

The causes of death were cardiac arrest, intracranial hemorrhage (ICH), and malignancy (cancer).

Table 2 compares some of the main characteristics of the sample between deceased and survived patients. The mean age of deceased patients was significantly higher than those survived. Most of the deceased patients were females. The duration of ventilation and hospitalization in the ICU were significantly higher in deceased patients. The scores of MSOFA 1, MSOFA 2, and MSOFA 3 as well as SOFA 1, SOFA 2, and SOFA 3 were significantly higher in the deceased patients.

### 3.2. Study Outcomes

**Figure 1:**
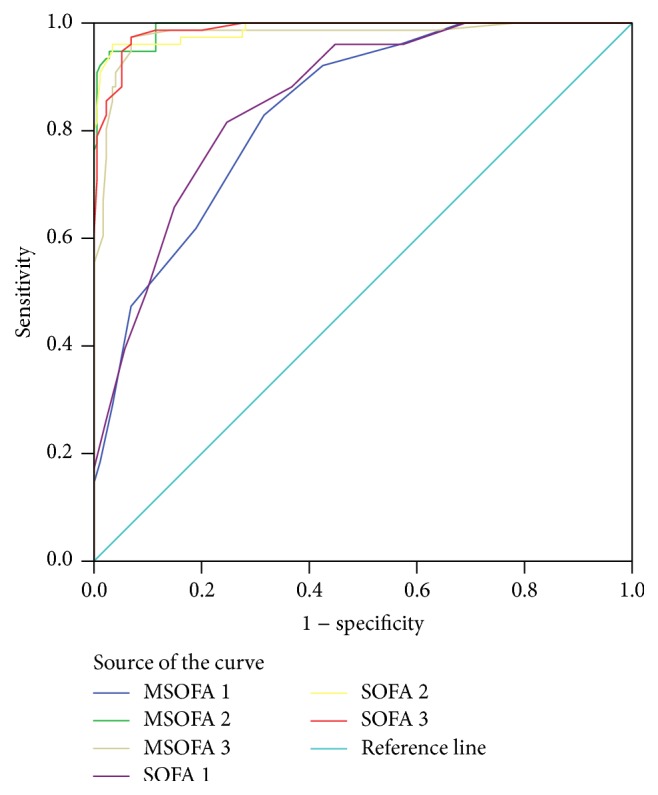
Comparing the predictive power SOFA 1, SOFA 2, and SOFA 3 and MSOFA 1, MSOFA 2, and MSOFA 3, in predicting mortality using ROC curve.

There was a significant positive correlation between the MSOFA 1 and SOFA 1 (*r*: 0.942, *P* < 0.0001), MSOFA 2 and SOFA 2 (*r*: 0.972, *P* < 0.0001), and also MSOFA 3 and SOFA 3 (*r*: 0.960, *P* < 0.0001). The performance of MSOFA and SOFA score is evaluated in Figure 1. Interpretation of the area under the ROC curve (AUC) showed that the performance of MSOFA 1 score with cut-off point of eight was AUC 0.837 (95% CI: 0.788–0.887). In predicting mortality, the sensitivity, specificity, positive predictive value (PPV), and negative predictive value (NPV) were 82.9%, 68.4%, 53.4%, and 90.2%, respectively.

The MSOFA 2 with the cut-off point of 9.5 (AUC: 0.992, 95% CI: 0.985–0.999; *P* < 0.0001) had 94.7%, 97.1%, 93.5%, and 97.7% sensitivity, specificity, positive predictive value (PPV), and negative predictive value (NPV) in predicting mortality, respectively.  Table 3 compares the area under the curve (AUC) for each tool with 95% CI.

## 4. Discussion

The main purpose of the present study was to compare the proposed MSOFA and original SOFA in predicting mortality rate of critically ill patients admitted to the ICU. Our results showed that both tools predict mortality with high accuracy. In the present study, the original SOFA and MSOFA scores predicted mortality as an excellent tool at the beginning, 24 hours after ICU admission, and 48 hours after ICU admission. We also found that proposed MSOFA 2 had a higher accuracy than other times. The ROC analysis and the area under the curve (AUC) were used to differentiate the power of a tool for distinguishing between survived and deceased patients. Based on the evidence AUC ≥ 0.7 shows acceptable tool and AUC ≥ 0.9 indicates excellent tool. Several studies have compared SOFA. In consistence with our results, Grissom et al. compared the modified SOFA (SPO_2_/FiO_2_ ratio instead of PaO_2_/FiO_2_ and jaundice in the sclera and body instead of measuring bilirubin and removal of platelets) with the original SOFA one. In their study, on the first day MSOFA and SOFA scores predicted mortality with AUC of 0.83 and AUC of 0.84 and on the third day with AUC of 0.78 and 0.79, respectively. They concluded that MSOFA could calculate mortality like original SOFA [16]. In another study, Oda et al. examined the MSOFA power to predict early mortality after Ventricular Assist Devices (VAD) placement. The MSOFA showed a good discrimination to determine the mortality risk of organ failure on the first 7 day with the maximum AUC of 0.99 (95% CI: 0.96–1.0). The MSOFA score more than 11 for detecting mortality had sensitivity of 92.9% and specificity of 96.7% [17].

In our study, the highest sensitivity (94.7%) and specificity (97.1%) of the MSOFA were on the second day (48 hr.) at the cut-off point of 9.5 to predict mortality. In addition, on the third day MSOFA (72 hr.) in the cut-off point of 9.3 showed sensitivity of 97.4% and specificity of 93.1% for predicting mortality. Similar to our results, Oda et al. concluded that the MSOFA has high accuracy to predict perioperative mortality [17]. Apart from the predicting mortality in the ICUs, SOFA has been used in several conditions for predicting morbidity. For example, Oliveira-Neto et al. evaluated the power of SOFA score to predict maternal morbidity and found that SOFA score could effectively evaluate the severity and estimating prognosis of morbidity [18]. In another study, Lin et al. suggested the modified model of SOFA for clinical assessment of patients with acute renal failure who require kidney transplantation [19]. Halim et al. compared the APACHE II, SOFA, and MSOFA to predict mortality in the ICU patients undergoing surgery. In consistence with our results, the mean scores of SOFA and MSOFA were significantly higher in deceased patients. They reported that the predictive power of the APACHE II was lower (AUC: 0.69; *P* < 0.001) than the predictive powers for the SOFA (AUC: 0.73; *P* < 0.001) and the initial MSOFA (AUC: 0.75; *P* < 0.001). Their results suggested that the mean and maximum SOFA and MSOFA scores are better indicators for predicting mortality in operated patients admitted to the ICU [14].

## 5. Limitations

When interpreting the results, the following study limitations have to be taken into account. First, a single center with partially small sample was used to compare the predictability of tools which places limitations on the quality of ICU care and external applicability. Therefore, a multicenter study would have given better external validity. The findings of our study need to be validated in a future prospective study. Second, we used a nonstandardized assessment of clinical parameters such as jaundice.

## 6. Conclusion

This study showed the excellent performance of the total MSOFA score in predicting patients mortality admitted in the ICU. The results of the present study demonstrated a strong positive correlation between the scores achieved from the original SOFA and the proposed MSOFA at the 24 hours, 48 hours, and 72 hours after ICU admission, and both tools are associated with high sensitivity and specificity for predicting mortality. Our proposed MSOFA due to lower cost and greater ease of application can be a proper alternative to the original SOFA. Therefore, we offer MSOFA as a more convenient and usable tool, particularly in the regions with limited resource.

## Figures and Tables

**Table 1:** Patients characteristics.

Properties	Categories	Values
Age (year)	Male 154 (61.6%)	44.39 ± 19.83 (ranged 18–48)
	Female 96 (38.4%)	48.50 ± 18.69 (ranged 18–82)

Cause of hospitalization	Traumatic	101 (40.4%)
	Nontraumatic	140 (59.4%)

History	Underlying disease	145 (58%)
	Prior history of hospitalization	33 (13.2%)
	Required intubation	164 (65.6%)
	Required tracheostomy	8 (3.2%)
	Required reintubation	4 (1.6%)

Patients morbidity/mortality outcomes	Brain death	2 (0.8%)
	Transfer to the other centers	7 (2.8%)
	Vegetative state	2 (0.8%)
	Quadriplegia in the ICU	1 (0.4%)
	Quadriplegia out of the ICU	2 (0.8%)
	Death	78 (31.2%)

**Table 2:** Comparison of demographic data, clinical findings, and SOFA-MSOFA scores.

Factors examined	Deceased patients *n* = 78 (31.2%)	Survived patients *n* = 172 (68.8%)	*P* value
Age (*M* of years ± SD)	51.29 ± 17.72	43.55 ± 19.78	0.003
Gender as *n* (%)			
Male	41 (26.62%)	113 (73.37%)	0.048
Female	37 (38.54%)	59 (61.46%)	
Duration of intubation (days)	1.05 ± 0.32	1.03 ± 0.23	0.691
Duration of ventilation (days)	9.22 ± 9.10	4.62 ± 6.12	0.048
Length of ICU stay (days)	7.96 ± 6.17	4.87 ± 4.86	<0.0001
Length of hospital stay (days)	9.24 ± 7.51	8.50 ± 6.09	0.41
MSOFA			
1	9.93 ± 3.17	5.98 ± 2.62	<0.0001
2	14.67 ± 3.88	5.19 ± 2.49	<0.0001
3	14.26 ± 3.88	5.54 ± 2.55	<0.0001
SOFA			
1	10.92 ± 3.06	6.31 ± 2.94	<0.0001
2	15.71 ± 3.60	5.51 ± 2.83	<0.0001
3	15.27 ± 3.30	5.98 ± 2.85	<0.0001

**Table 3:** Comparison of predictive power of SOFA 1, SOFA 2, and SOFA 3 and MSOFA 1, MSOFA 2, and MSOFA 3, in predicting mortality.

Tools	AUC^*∗*^	Confidence interval of 95%		*P* value
		Lower bound	Upper bound	
MSOFA				
1	0.837	0.788	0.887	<0.0001
2	0.992	0.985	0.999	<0.0001
3	0.977	0.957	0.988	<0.0001
SOFA				
1	0.857	0.811	0.904	<0.0001
2	0.988	0.977	1.000	<0.0001
3	0.988	0.978	0.997	<0.0001

^*∗*^AUC: area under curve.
